# Peptides Derived from a Phage Display Library Inhibit Adhesion and Protect the Host against Infection by *Paracoccidioides brasiliensis* and *Paracoccidioides lutzii*

**DOI:** 10.3389/fphar.2016.00509

**Published:** 2016-12-23

**Authors:** Haroldo C. de Oliveira, Jussara S. Michaloski, Julhiany F. da Silva, Liliana Scorzoni, Ana C. A. de Paula e Silva, Caroline M. Marcos, Patrícia A. Assato, Daniella S. Yamazaki, Ana M. Fusco-Almeida, Ricardo J. Giordano, Maria J. S. Mendes-Giannini

**Affiliations:** ^1^Universidade Estadual Paulista (UNESP), Faculdade de Ciências Farmacêuticas, Câmpus Araraquara, São PauloBrasil; ^2^Universidade de São Paulo (USP), Instituto de Química, Câmpus São Paulo, São PauloBrasil

**Keywords:** adhesion, anti-adhesive peptides, phage display, *Paracoccidioides* spp., paracoccidioidomycosis

## Abstract

*Paracoccidioides brasiliensis* and *Paracoccidioides lutzii* are dimorphic fungi and are the etiological agents of paracoccidioidomycosis (PCM). Adhesion is one of the most important steps in infections with *Paracoccidioide*s and is responsible for the differences in the virulence of isolates of these fungi. Because of the importance of adhesion to the establishment of an infection, this study focused on the preliminary development of a new therapeutic strategy to inhibit adhesion by *Paracoccidioides*, thus inhibiting infection and preventing the disease. We used two phage display libraries to select peptides that strongly bind to the *Paracoccidioides* cell wall to inhibit adhesion to host cells and extracellular matrix (ECM) components (laminin, fibronectin, and type I and type IV collagen). This approach allowed us to identify four peptides that inhibited up to 64% of the adhesion of *Paracoccidioides* to pneumocytes *in vitro* and inhibited the adhesion to the ECM components by up to 57%. Encouraged by these results, we evaluated the ability of these peptides to protect *Galleria mellonella* from *Paracoccidioides* infection by treating *G. mellonella* larvae with the different peptides prior to infection with *Paracoccidioides* and observing larval survival. The results show that all of the peptides tested increased the survival of the larvae infected with *P. brasiliensis* by up to 64% and by up to 60% in those infected with *P. lutzii*. These data may open new horizons for therapeutic strategies to prevent PCM, and anti-adhesion therapy could be an important strategy.

## Introduction

Paracoccidioidomycosis (PCM) is a systemic mycosis caused by the dimorphic fungi *Paracoccidioides brasiliensis* and *Paracoccidioides lutzii* that occurs in Latin America and in Brazil, which is the country with the largest number of endemic areas for this disease in the world. The incidence of the disease in endemic areas has been estimated to be approximately one to three clinical cases per 100,000 inhabitants per year (Coutinho et al., 2002), and it is an important public health issue.

An infection with *Paracoccidioides* spp. begins with the adhesion of fungal cells to host cells, which is mediated by a special class of proteins present on the cell wall known as adhesins (Silva, 2008). The pathogen’s ability to colonize and invade the host tissue is strictly dependent on these proteins and the adhesion capacity of the fungus. de Oliveira et al. (2015), using *in vivo* animal models, demonstrated that the ability to express adhesins and adhere to the host are decisive factors in the virulence of different *Paracoccidioides* spp. isolates.

The inhibition or blocking of fungal adhesion to host cells may be an innovative and efficient way to prevent infection. Such strategies would reduce fungal colonization of different host tissues and nutrient acquisition and, consequently, facilitate the actions of the host immune system to fight the infection. This type of therapy is known as anti-adhesion therapy and can be an effective way to improve the efficacy of PCM treatments. In support of this idea, de Oliveira et al. (2015) demonstrated that when blocking two important *Paracoccidioides* spp. adhesins, enolase and 14-3-3, using specifics antibodies, host organisms were able to resist fungal infection and showed increased survival.

Anti-adhesive compounds can competitively inhibit adhesion by mimicking either microbes or host cell ligands. Alternatively, antibodies that recognize the surface epitopes on the pathogen can be used to block adhesion and, in the process, can actively or passively immunize the host (Krachler and Orth, 2013).

Resistance to anti-adhesive agents may also be expected to emerge. Resistance to drugs arises spontaneously in a population through mutations. The persistent use of antibiotics will result in the death of all non-resistant bacteria. Therefore, only those with mutations conferring resistance can propagate, resulting in the quick spread of resistance in a population. However, unlike with the use of antibiotics, which kill or stop the growth of susceptible microorganisms, during anti-adhesion therapy, nonresistant strains can continue to propagate and be transmitted to new hosts. Any wild-type strains would continue to compete with resistant strains in untreated individuals. This would potentially allow sensitive and resistant organisms to propagate and be transmitted at equivalent rates, dramatically slowing the emergence of a predominantly resistant population (Ofek et al., 2003; Cozens and Read, 2012).

Phage display has been extremely important for identifying and characterizing new high-affinity ligands and their receptors in the context of specific diseases. It has also been helpful for the identification of molecules with different applications, from diagnostic biomarkers to potential targets for use in the treatment of different infections (Lionakis et al., 2005; Antonara et al., 2007; Mullen et al., 2007; Shkoporov et al., 2008; Posadas et al., 2012), since the selected peptides frequently have biological activity related to the nature of the molecule or cell in a study.

These characteristics of phage display allow for the identification of therapeutic targets relevant to many biological processes in an organism and simultaneously allow for the isolation and characterization of peptide antagonists or agonists for specific targets. Therefore, peptides isolated via phage display can be exploited for the development of novel therapeutic agents for use in rational drug design, targeted therapy, gene therapy, vaccine production, diagnostic testing, and other applications (Sergeeva et al., 2006; Giordano et al., 2009).

This study proposed the use of the phage display technique to select peptides that interacted with molecules and receptors involved in the interaction of *Paracoccidioides* spp. with host and in that way, evaluated these selected peptides as candidates for anti-adhesive molecules to be a new alternative therapy against PCM.

## Materials and Methods

### *Paracoccidioides* Isolates

Two *Paracoccidioides* isolates were used: Pb18 (*P. brasiliensis*) and Pl01 (*P. lutzii*), maintained *in vitro* in Fava Netto’s medium at 37°C (Fava Netto, 1961). The handle of *Paracoccidioides* spp. cultures was made in a Class II Biological Safety Cabinet according recommendations of American Type Culture Collection (ATCC) and Brazilian Ministry of Health, Secretariat of Science, Technology and Strategic Inputs (Ministério da Saúde and Estratégicos, 2006).

### Phage Display Peptides Libraries X6 and CX8C

Phage libraries were produced using the vector fUSE55 and the methodology developed by George Smith (Smith and Scott, 1993), with modifications to improve the efficiency and the number of final clones (Koivunen et al., 1993; Koivunen et al., 1999). The libraries were produced and provided by Dr. Ricardo José Giordano from the Laboratory of Vascular Biology, University of Sao Paulo (USP) and were X6 (a linear library) and CX8C (a cyclic library; X represents any amino acid, and C = cysteine that allows for cyclical peptides) libraries (Michaloski et al., Submitted).

### Selection of Peptides That Bind to *P. brasiliensis*

The BRASIL (**B**iopanning and **R**apid **A**nalysis of **S**elective **I**nteractive **L**igands) technique (Giordano et al., 2001) was used for the selection of peptides that bind to *Paracoccidioides* spp. This technique consists of the selection of phages that bind to live cells and the use of a two-phase system to separate the cells and phages in a single centrifugation step. First, 10^9^ transduction units (TU) of each phage display library were separately incubated with *Saccharomyces cerevisiae* (S288C) (10^6^ cells) in 100 μl of PBS. After 3 h on ice, the suspensions containing the phages and the cells were transferred to the top of a nonmiscible organic lower phase known as BRASIL oil [dibutyl phthalate:cyclohexene (9:1) v/v] and centrifuged (10,000 × *g*, 10 min, 25°C). After centrifugation, the supernatants containing phages that did not bind to *S. cerevisiae* were recovered and transferred to a suspension of 10^6^ Pb18 cells. After 3 h on ice, the cell suspensions were again transferred on to BRASIL oil and centrifuged at 10,000 × *g* for 10 min. The pellets of Pb18 cells that formed at the bottom of the organic phase were collected, and the phages bound to the cells were recovered via infection with log-phase *E. coli* K91 in LB medium supplemented with kanamycin and tetracycline (100 μg/mL and 20 μg/mL, respectively) and incubated at 37°C, with rotation at 250 rpm, for 20 h. The amplified phages were recovered from the culture medium via precipitation with polyethylene-glycol/NaCl and were used for subsequent rounds of selection (second and third) to further enrich for phages from each library that bound to Pb18 cells.

### Binding Assays

*Paracoccidioides brasiliensis*, *P. lutzii*, and *S. cerevisiae* cells were incubated with 10^9^ TU of the phage to be tested, as described above. After 2 h of incubation on ice, they were transferred to the top of BRASIL oil as previously described in the item 2.4 and centrifuged (10,000 × *g*, 10 min, 25°C). The pellet was placed in a new tube, and the phages were recovered via infection with *E. coli* K91. Serial dilutions were seeded onto LB/Kan/Tet plates to quantify the number of phage bound to each receiver. As a control *P. brasiliensis* and *P. lutzii* cells were incubated with 10^9^ of the insertless phage Fd-tet. Three independent experiments were performed.

### Inhibition of *Paracoccidioides* spp. Adhesion to Extracellular Matrix (ECM) Components by the Selected Peptides Using Enzyme Linked Immunosorbent Assay (ELISA)

Ninety-six-well plates were separately pre-coated with 10 μg/mL of each tested ECM component (laminin, fibronectin, and type I and type IV collagen) in a 0.05 M carbonate buffer (pH 9.6) at room temperature for 1 h. The plates were then washed with PBS/Tween-20, 0.05% (PBS-T) and blocked with 1% bovine serum albumin (BSA) for 2 h at 37°C. The two *Paracoccidioides* isolates (Pb18 and Pl01, 10^6^ cells/mL) were incubated with 200 μg/mL of each studied peptide, which were synthesized by the Chinese Peptide Company (China), at 37°C, with rotation at 250 rpm, for 1 h. These inocula were incubated with the ECM components for 15 h at 37°C. The plates were washed with PBS-T, and an anti-cell free *Paracoccidioides* antibody (1:100 in PBS-T and 0.5% BSA) (Blotta and Camargo, 1993) was added for 1 h at 37°C. Another wash cycle was performed, and a secondary antibody, anti-rabbit IgG-HRP (1:2000 in PBS-T and 0.5% BSA), was added for 1 h at 37°C. The colorimetric reaction was observed by adding a solution of 0.2 M sodium phosphate, 0.1 M citric acid, 0.4 mg/mL o-phenylenediamine, and 30% hydrogen peroxide for 10 min, followed by the addition of 3 M hydrochloric acid. Absorbance was measured at 490 nm and converted to percent inhibition. For this conversion, *Paracoccidioides* spp. cells without treatment were used as a control, and the absorbance of this control was set as 100% adhesion. The difference in the absorbance in the treated *Paracoccidioides* spp. cells relative to absorbance in the control was considered the percentage of adhesion inhibition. This experiment was performed in triplicate, with three independent experiments.

### Inhibition of *Paracoccidioides* spp. Adhesion to A549 Pneumocytes Cell Line by the Selected Peptides Using ELISA

The continuous culture of A549 cells line took place in F-12 Nutrient Mixture (HAM-F12) medium supplemented with 10% fetal bovine serum (FBS) in sterile plastic bottles maintained at 36.5°C with 5% CO_2_. After 3–4 days, the bottles were trypsinized by washing the cell monolayer with 1 mL of a 0.2% trypsin ATV solution (Adolfo Lutz) supplemented with 0.02% EDTA. After 1–2 min, the cells were homogenized with variable volumes of HAM-F12 medium supplemented with 10% of FBS to neutralize the trypsin. The total volume of the cell suspension was determined to achieve a concentration of 10^6^ cells/mL. For the assay, 10^5^ A549 pneumocytes were added to each well of a 96-well plate and then infected over 15 h (37°C with 5% CO_2_) with the different *Paracoccidioides* isolates (Pb18 and Pl01, 10^6^ cells/mL) that were previously incubated with 200 μg/mL of each studied peptide at 37°C for 1 h, with rotation at 250 rpm. After incubation, ELISAs were performed as described in the Section “Inhibition of *Paracoccidioides* spp. Adhesion to Extracellular Matrix (ECM) Components by the Selected Peptides Using Enzyme Linked Immunosorbent Assay (ELISA).” In this experiment, untreated *Paracoccidioides* spp. cells were also used as controls. This experiment was performed in triplicate, with three independent experiments.

### Determination of the Antifungal Activity of the Peptides

The minimum inhibitory concentration (MIC) of each peptide was determined following the standardized method in the M27-A3 document CLSI (2008). Amphotericin B (Sigma) was used as a control, and the final concentrations of peptides used ranged from 400 to 0.008 μg/mL after the addition of the inocula. Both peptides and amphotericin B were prepared with RPMI 1640 medium (Gibco) supplemented with 2% glucose (RPMI 1640-2%). The fungal cells were suspended in PBS, and the number of viable cells was estimated by staining them with Trypan blue and counting them with a haemocytometer. A suspension with 10^6^ cells/mL was diluted at 1:50 in PBS, and then was diluted at 1:20 in RPMI 1640-2% Glc. The plates were incubated at 35°C, with rotation at 150 rpm, for 48 h, and the MABA (Microplate AlamarBlue Assay) was employed. Samples were incubated for an additional 24 h, for a total of 72 h for the final determination of MIC values, according de Paula e Silva et al. (2013). Three independent experiments were performed.

### Determination of the Cytotoxicity of the Peptides Using the MTT (3-(4,5-Dimethylthiazol-2-yl)-2,5-Diphenyltetrazolium Bromide) Cell Viability Test

The cytotoxicity of the selected peptides was determined using the A549 pneumocyte linage. The peptides were evaluated at concentrations ranging from 400 to 25 μg/mL using the MTT cell viability test according Denizot and Lang (1986) and Ferrari et al. (1990). For this, plates were prepared with 10^6^ cells/well in HAM-F12 medium. Before the experiments, the medium was removed, and 100 μL of each tested dilution of the peptides in HAM-F12 was placed in contact with the cells. The plates were incubated at 37°C under 5% CO_2_ in the dark for 24 h. The solutions were then removed, 10 μL of MTT solution (5 mg/L) was added, and the plates were incubated again at 37°C for 4 h. After incubation, the MTT solution was removed, 100 μl of isopropanol was added to dissolve the precipitate, and the plates were read spectrophotometrically at 595 nm DO. The dead control was 10% hydrogen peroxide. The live control was untreated cells, and the reading from this control was considered as 100% living cells. This experiment was performed in triplicate, with three independent experiments, and the percentage of viable cells was calculated using the following formula:

%Viable cells = mean of the test×100/mean of the negative control.

### *In vivo* Toxicity of the Peptides in *Galleria mellonella*

The toxicity of the peptides was evaluated using 2, 4, 8, 50, 100, 200, and 400 μg/larva of each peptide. For this assay, groups of 16 larvae were treated with the various concentrations tested and incubated at 37°C. The survival of the larvae was evaluated every day for 7 days after treatment. As controls, we used a group of larvae treated with PBS and a group of untreated larvae. Three independent experiments were performed.

### Capacity of Peptides to Prevent the Infection of *G. mellonella* with *Paracoccidioides* spp.

Larval groups (*n* = 16) were separately treated with 100 μg/larvae of each peptide for 3 h. The larvae were subsequently infected with 5 × 10^6^ cells/larva of *Paracoccidioides* spp. isolates (Pb18 or Pb01) according to Scorzoni et al. (2015), and larval survival was then observed daily for 1 week. The peptide treatments and fungal inoculations were performed with 10 μL Hamilton syringes (Hamilton, USA). Larval death was evaluated based on the visual confirmation of a lack of movement after the larvae were touched with tweezers. As controls, groups of larvae treated with PBS and infected with Pb18 or Pb01 and a group of uninfected larvae only treated with PBS were used. Three independent experiments were performed.

### Effect of Peptide Treatment on Haemocyte Density

Groups of 16 larvae were inoculated with 100 μg/larvae of each peptide and incubated at 37°C for 3 h. The peptide inoculations were performed with 10 μL Hamilton syringes (Hamilton, USA). After incubation, the haemolymph from each larva was collected separately and diluted 1:10 in PBS. The cells were counted in a haemocytometer under a bright field microscope. A group treated with PBS was used as the control. Three independent experiments were performed.

### Statistical Analysis

Statistical analyses and graph design were performed using GraphPad Prism version 5.00 (GraphPad Software, San Diego, CA, USA), and the results were considered significant when *p* < 0.05. Binding assay, MTT test and the haemocyte density test results were analyzed using one-way ANOVAs and Tukey’s *post hoc* tests. Survival assays were analyzed using Mantel–Cox log-rank tests.

## Results

### Selection of Peptides That Bound to the Surface of *P. brasiliensis* Cells via Phage Display Using BRASIL Technique

We employed the BRASIL technique to select peptides that bound to *P. brasiliensis* using linear (X6) and cyclic (CX8C) peptide libraries of different sizes to increase the variety of peptides available for study. A pre-clearing step was added to our selection protocol to favor the selection of peptides that specifically bound to this species. Phage libraries were first incubated with the non-pathogenic yeast *S. cerevisiae* to remove ubiquitously binding peptides. The remaining unbound phages were transferred to the pathogenic *P. brasiliensis* strain, and bound phages were recovered for further rounds of selection. After three rounds of selection, we observed an enrichment of phages bound to *P. brasiliensis* compared to the second round (2.3 times for the X6 library and five times for CX8C library), indicating an increase in phages that could bind to the fungal cells with a high affinity.

At the end of the selection process, 96 individual phage clones from each library were randomly picked from the third round and sequenced, and 84 and 76 sequences were obtained from the X6 and CX8C libraries, respectively. Four peptides were selected because they were displayed by multiple phages (**Table 1**): three peptides from the X6 library and one from CX8C library. These peptides were selected for further study because they were likely to have higher affinities for *P. brasiliensis* cells.

**Table 1:** Peptide sequences and frequency.

Peptides sequences	*N*	Percentage (%)
LVGRVV	5	5.9
LDFVVG	5	5.9
CSVSALGGAC	3	3.9
VVAGSV	3	3.5


*Selected peptides amino acids sequences. The other sequences obtained from both libraries demonstrated a frequency of 1.3% or less and because of this were not selected for further studies.*

### Binding Assays

**FIGURE 1:**
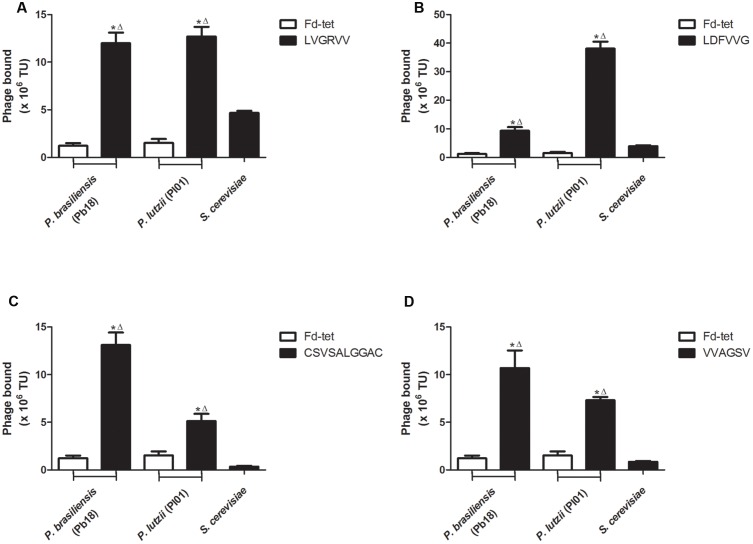
**Selected peptides bind to *Paracoccidioides* cells.** Binding assays of the four selected phages to *Paracoccidioides brasiliensis* (Pb18), *Saccharomyces cerevisiae*, and *Paracoccidioides lutzii* (Pl01) isolates. Binding of LVGRVV **(A)**, LDFVVG **(B)**, CSVSALGGAC **(C)**, and VVAGSV **(D)** phages. Binding of the control phage Fd-tet to *P. brasiliensis* (Pb18) and *P. lutzii* is indicated. TU, transduction units. ^∗^*p* < 0.05 (compared with Fd control); Δ*p* < 0.05 (compared with the *S. cerevisiae* control).

We evaluated the binding of each peptide to *P. brasiliensis* and *P. lutzii* and to the non-pathogenic yeast *S. cerevisiae*. As a control, we evaluated the binding of the insertless phage Fd-tet to *P. brasiliensis* and *P. lutzii* cells. We observed that all peptides showed greater binding to *P. brasiliensis* (phage LVGRVV, 9.7-fold; phage LDFVVG, 7.5-fold; phage CSVSALGGAC, 10.5-fold; and phage VVAGSV, 8.1-fold) and *P. lutzii* (phage LVGRVV, 9.7-fold; phage LDFVVG, 30.8-fold; phage CSVSALGGAC, 4.1-fold; and phage VVAGSV, 5.9-fold) compared with the binding of the Fd-tet control phage (**Figure 1**). None of the phages tested were found to significantly bind to *S. cerevisiae*, indicating that the pre-clearing step efficiently removed the peptides that bound to these cells. In summary, our validation assays confirmed that all selected peptides bound to specific cell surface ligands of the two species of the genus *Paracoccidioides*, *P. brasiliensis* (isolate Pb18) and *P. lutzii* (isolate Pl01).

### Inhibition of *Paracoccidioides* spp. Adhesion to ECM Components and A549 Pneumocytes by the Selected Peptides Using an Inhibition Enzyme Linked Immunosorbent Assay (inh-ELISA)

**FIGURE 2:**
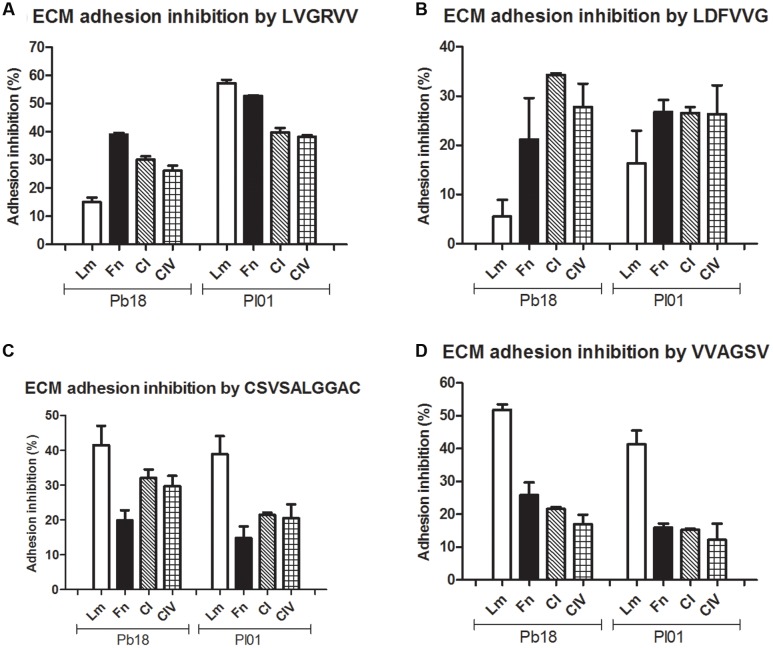
**Inhibition of *P. brasiliensis* and *P. lutzii* adhesion by the selected peptides to extracellular matrix (ECM) components laminin, fibronectin, type I and type IV collagen tested via inhibition ELISAs.** **(A)** Inhibition by LVGRVV, **(B)** inhibition by LDFVVG, **(C)** inhibition by CSVSALGGAC, and **(D)** inhibition by VVAGSV.

**FIGURE 3:**
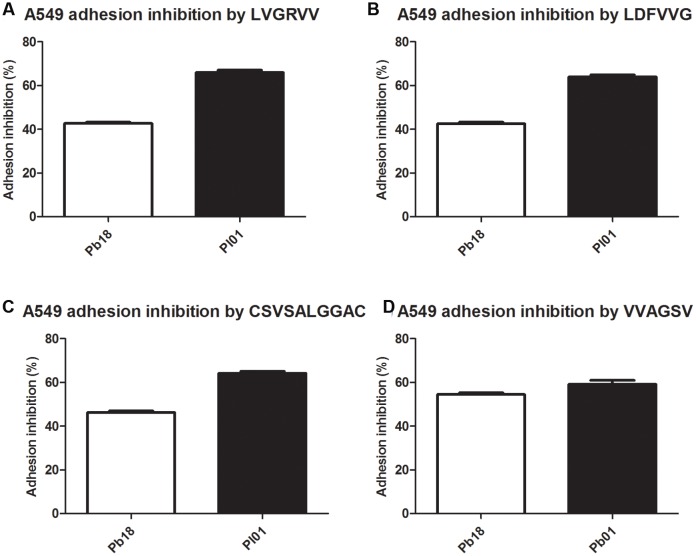
**Inhibition of *P. brasiliensis* and *P. lutzii* adhesion to A549 cells by the selected peptides tested via inhibition ELISAs.** **(A)** Inhibition by LVGRVV, **(B)** inhibition by LDFVVG, **(C)** inhibition by CSVSALGGAC, and **(D)** inhibition by VVAGSV.

Assays were conducted using four different ECM components (laminin, fibronectin, and collagen types I and IV) (**Figure 2**) and the A549 pneumocyte line (**Figure 3**). The results indicate the strong potential of the peptides to inhibit the adhesion of *P. brasiliensis* and *P. lutzii* and indicate that these peptides might bind to some components of the fungal cell wall important for the *Paracoccidioides-*host interaction.

All the peptides tested inhibited the adhesion to all of the ECM components, with inhibition ranging from 10 to 60% depending on the strain and the ECM component (**Figure 2**). LVGRVV more efficiently inhibited the adhesion of *P. brasiliensis* to fibronectin (39%) and the adhesion of *P. lutzii* to laminin (57%). LDFVVG efficiently inhibited the adhesion of both fungi to fibronectin and type I and type IV collagen (from 21 to 34% inhibition). CSVSALGGAC and VVAGSV efficiently inhibited the adhesion of both fungi to laminin, with an inhibition rate ranging from 38 to 51%.

All tested peptides were able to inhibit the adhesion of *P. brasiliensis* and *P. lutzii* to A549 pneumocytes in a similar manner. The inhibition of the adhesion of the Pb18 and Pl01 strains to A549 pneumocytes ranged from 40 to 60%, respectively, for all the studied peptides (**Figure 3**).

### Determination of the Antifungal Activities of the Peptides

The peptides did not kill or inhibit the growth of *P. brasiliensis* and *P. lutzii* at any tested concentration, which ranged from 400 to 0.008 μg/mL (data not shown).

### Determination of the Cytotoxicity of the Peptides Using the MTT Cell Viability Test

The peptides did not show cytotoxicity. The percentage of viable cells was over 92% for LVGRVV and LDFVVG and was 100% for CSVSALGGAC and VVAGSV at a concentration of 200 μg/mL, which was the concentration used in the inh-ELISA. All concentrations lower than 100 μg/mL showed no cytotoxicity (data not shown).

### Toxicity of Peptides in *G. mellonella*

No larvae died at any concentration tested, demonstrating that the peptides are nontoxic to the larvae. These results complemented the *in vitro* tests in which the peptides showed no toxicity to A549 pneumocytes.

### Protection of *G. mellonella* from Infection with *P. brasiliensis* and *P. lutzii* by the Peptides

**FIGURE 4:**
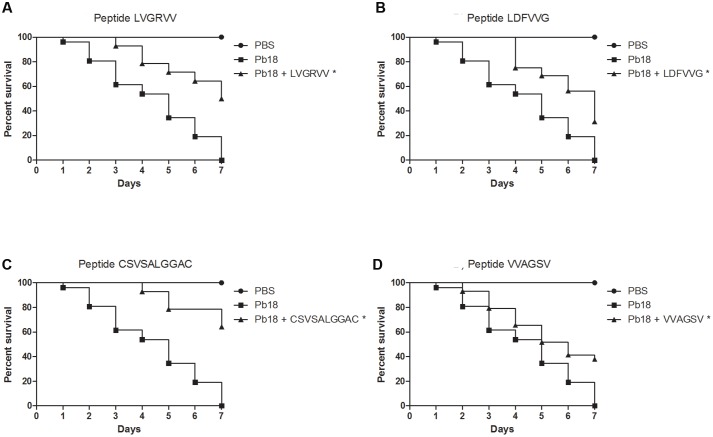
**Capacity of selected peptides to protect *G. mellonella* larvae against *P. brasiliensis* infection.** The graphs show the percentage of *G. mellonella* larvae treated with the selected peptides and challenged with *P. brasiliensis*. **(A)** Treatment with LVGRVV, **(B)** treatment with LDFVVG, **(C)** treatment with CSVSALGGAC, and **(D)** treatment with VVAGSV. ^∗^Statistical significance (*p* < 0.05) is relative to Pb18 (infected and untreated larvae).

All peptides significantly (*p* < 0.05) protected the larvae against *P. brasiliensis* infections (**Figure 4**). Survival rates of 50%, 31%, 64%, and 37% were observed for larvae treated with the LVGRVV, LDFVVG, CSVSALGGAC, and VVAGSV peptides, respectively. The survival time increased when the larvae were treated with each of the peptides. Untreated larvae began to die after 1 day, but the treated larvae began to die after 3 days for LVGRVV, 4 days for LDFVVG and CSVSALGGAC and 2 days for VVAGSV, and in all cases, 7 days was not sufficient to kill all treated larvae, whereas 7 days were sufficient to kill all untreated larvae.

**FIGURE 5:**
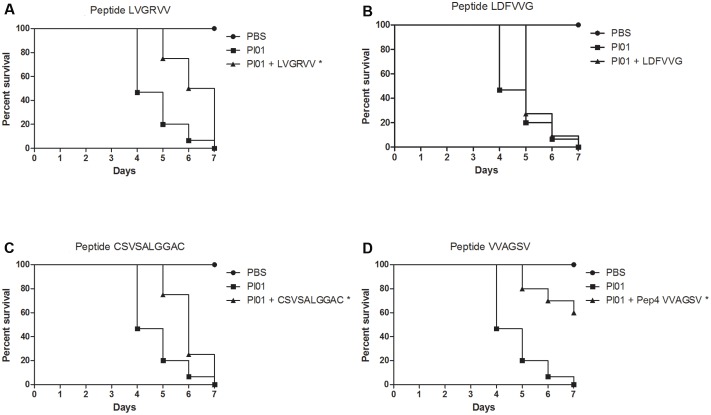
**Capacity of the selected peptides to protect *G. mellonella* larvae against *P. lutzii* infection.** The graphs show the percentage of *G. mellonella* larvae treated with the selected peptides and challenged with *P. lutzii*. **(A)** Treatment with LVGRVV, **(B)** treatment with LDFVVG, **(C)** treatment with CSVSALGGAC, and **(D)** treatment with VVAGSV. ^∗^Statistical significance (*p* < 0.05) is relative to Pl01 (infected and untreated larvae).

**Figure 5** shows the results obtained for peptide-treated larvae infected with *P. lutzii*. Only the VVAGSV peptide was able to significantly (*p* < 0.05) protect the larvae against *P. lutzii* infections, resulting in a survival rate of 60% at the end of the seventh day of infection. However, the LVGRVV and CSVSALGGAC peptides were able to significantly (*p* < 0.05) increase the survival time. Treatment with LDFVVG did not affect larval survival after *P. lutzii* infection. The survival time increased when the larvae were treated with each peptide. Untreated larvae began to die after 4 days, whereas larvae treated with the different peptides began to die after 5 days. After treatment with the VVAGSV peptide, 7 days were not sufficient to kill all of the larvae (**Figure 5**).

### Determination of Haemocyte Density after Treatment with the Selected Peptides

**FIGURE 6:**
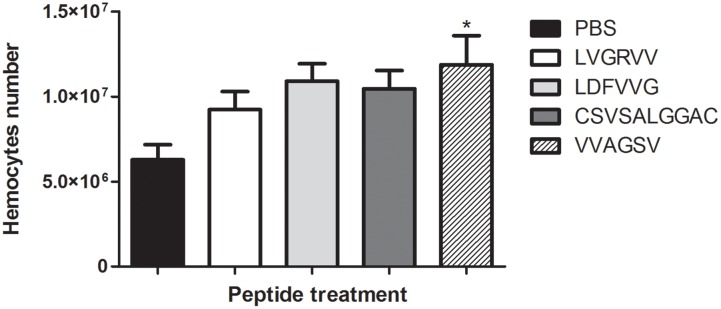
**Haemocyte density.** The graph shows alterations in haemocyte density in the different *G. mellonella* larvae groups after 3 h of treatment with LVGRVV, LDFVVG, CSVSALGGC, and VVAGSV peptides. ^∗^*p* < 0.05.

Larvae were treated with the selected peptides for 3 h, and we observed that peptide VVAGSV stimulated significantly the haemocyte production (*p* < 0.05) compared with production in the PBS control (**Figure 6**).

## Discussion

*Paracoccidioides* spp. express various adhesins, and the adhesion mediated by these molecules is the most important step in a successful infection. Therefore, inhibiting adhesion to host cells may be an interesting way to protect the host from *Paracoccidioides* infections. The use of molecules that interfere with the adhesion of pathogens, known as anti-adhesion therapy, appears to be an efficient way to prevent infection because if the fungus cannot adhere, it is incapable of colonizing the host tissue, and its acquisition of nutrients is impeded, which facilitates resistance by the host’s immune system.

In this study, four peptides selected by phage display promoted the *in vitro* inhibition (up to 57%) of *P. brasiliensis* and *P. lutzii* adhesion to different ECM components and to A549 pneumocytes (up to 64%). These results indicate that the anti-adhesive properties of these peptides could prevent the adhesion of *Paracoccidioides* spp. We demonstrated that these peptides did not have antifungal activity, which means that the reduction in the adhesion of both fungi was not caused by a loss of fungal viability during the experiments. In addition, the cytotoxicity of these peptides was tested using the MTT test, and no cytotoxicity was observed in A549 pneumocytes or *in vivo* in *G. mellonella*. These results indicate the possible safe therapeutic use of these substances.

We preliminarily tested the efficiency of these peptides to protect the host against *Paracoccidioides* infections in *G. mellonella*, an alternative animal model for pathogenicity and virulence studies on fungal pathogens (Brennan et al., 2002; Slater et al., 2011) that has recently been used to study the pathogenicity and virulence of *Paracoccidioides* spp. (de Oliveira et al., 2015; Marcos et al., 2015; Scorzoni et al., 2015).

The treatment of *G. mellonella* prior to *P. brasiliensis* infection demonstrated that the peptides were able to prevent larval death for up to 7 days after infection, with survival rates of 50% (LVGRVV), 31% (LDFVVG), 64% (CSVSALGGAC), and 37% (VVAGSV), and treatments increased the survival time of the larvae. For *P. lutzii*, only the VVAGSV peptide increased the survival rate of larvae after infection, to 60%; however, the LVGRVV and CSVSALGGAC peptides were able to significantly increase larval survival times. These results revealed that all peptides have the potential to prevent *Paracoccidioides* spp. infection. We also observed that treatment with the peptides led to an increase in larval survival times when infected with both fungi. We believe that the peptides bind to molecules on the fungal cells important for adhesion, obstructing infection by the fungus. Furthermore, these peptides might compete with the fungus for binding sites on the host cell, also inhibiting fungal adhesion and facilitating the ability of the host to resolve the infection or making the infectious process difficult for the fungus. These characteristics show us that all the selected peptides are potential anti-adhesive therapeutic agents.

The stimulation of the immune response to counter the undesirable effects caused by the disease and reduce fungal burden are objectives to be explored for the effective treatment of severe PCM cases (Travassos and Taborda, 2012). However, since PCM primarily affects rural workers, the disease is associated with significant social and economic factors that mostly affect less affluent populations (Shikanai-Yasuda et al., 2006; de Amorim et al., 2013), so the development of new molecules that could work as a vaccine directed at high-risk populations could be an important step in effective prophylaxis for this disease.

The body cavity of *G. mellonella* contains haemolymph, which has functions similar to those of blood in mammals, carrying nutrients and signal molecules and participating in gas exchange (Bergin et al., 2003). Additionally, haemolymph contains cells known as haemocytes and antimicrobial peptides that can immobilize and kill invading microbes (Cotter et al., 2000).

We observed that when larvae were treated with the VVAGS peptide, haemocyte density increased (*p* < 0.05). These results suggest that this peptide can modulate the immune system of the larvae, increasing haemocyte production. This appeared to trigger a protective effect in the host, resulting in the protection of the larvae against *Paracoccidioides* spp. infections, which is important in anti-fungal therapy.

A peptide known as p10, derived from the *P. brasiliensis* adhesin gp43, has been shown to be recognized by the T lymphocytes of mice and humans and has a protective effect, and isogenic mice developed a 200-fold less intense infection when immunized with this peptide (Taborda et al., 1998). Furthermore, a combination of p10 immunization with various antifungal drugs showed an additive protective effect, with a significant reduction in CFU counts after 60 and 120 days of infection. Histological analyses revealed little or no *P. brasiliensis* cells in mouse tissue, and the use of p10 combined with drugs improved the standard therapy with antifungal drugs and reduced the duration of treatment (Marques et al., 2006). This type of combined therapy was also effective in anergic mice, and anergy is a common in patients suffering from acute and sub-acute PCM (Marques et al., 2008). Currently, studies using a DNA vaccine coding for p10 have shown satisfactory results; the administration of the vaccine 1 month before or after infection induced a significant reduction in the fungal burden in the lungs of mice, indicating that immunization with this vaccine is a powerful tool for the prevention and treatment of PCM (Rittner et al., 2012).

The selected peptides demonstrated anti-adhesive activity, inhibiting the adhesion of *P. brasiliensis* and *P. lutzii* to pneumocytes and ECM components. The treatment of infected *G. mellonella* with the peptides increased larval survival and increased haemocyte density, demonstrating a possible immunomodulatory effect in the larvae that can affect their survival. More studies are necessary to understand the mechanisms involved during the use of these peptides, including studies with murine models and studies focused on the combined use of the peptides and traditional antifungal therapies. However, our study shows that these molecules might provide new insights on the treatment and prevention of PCM.

## Author Contributions

HdO, AF-A, RG, and MM-G conceived and designed the experiments. HdO, JM, JdS, LS, AS, CM, PA, and DY performed the experiments. HdO, JM, JdS, LS, AS, CM, PA, DY, AA, RG, and MM-G analyzed the data. HdO, RG, and MM-G drafted the manuscript. All the authors read and approved the final manuscript.

## Conflict of Interest Statement

The authors declare that the research was conducted in the absence of any commercial or financial relationships that could be construed as a potential conflict of interest.
